# Injectable Hydroxyapatite-Reinforced Methacrylated Recombinant Type III Collagen Microgels for Soft-Tissue Filling

**DOI:** 10.3390/gels12090762

**Published:** 2026-08-26

**Authors:** Qianqian Zhu, Cuicui Wu, Xi Luo, Shihao Dong, Beijuan Luo, Changcheng Yin, Zhuangzhuang Cai, Shunqing Tang

**Affiliations:** Key Laboratory of Biomaterials of Guangdong Higher Education Institutes, Department of Biomedical Engineering, College of Life Science and Technology, Jinan University, Guangzhou 510632, China; zqq18528187200@163.com (Q.Z.); 15938642823@163.com (C.W.); khdwuen@163.com (X.L.); 19925716770@163.com (S.D.); lbj895570402@163.com (B.L.); 13437638873@163.com (C.Y.); 13600095217@163.com (Z.C.)

**Keywords:** recombinant type III collagen, methacryloylation, hydroxyapatite, microgel, dermal filling

## Abstract

Injectable fillers that combine immediate volume restoration with a sustained biological response remain of considerable interest in minimally invasive aesthetic medicine. In this study, a hydroxyapatite-loaded methacrylated recombinant type III collagen microgel (HAp@rhCol III-MA) was prepared by in situ coprecipitation and photocrosslinking. Recombinant type III collagen (rhCol III) was functionalized with methacryloyl groups to obtain rhCol III-MA, after which a calcium phosphate phase was mineralized in the presence of the modified collagen and the collagen phase was crosslinked under ultraviolet irradiation. More than 80% of the microgel particles prepared at 900 rpm were 20–80 μm in diameter. Spectroscopic, elemental, thermal, and diffraction analyses supported the incorporation of a poorly crystalline, HAp-compatible calcium phosphate phase, while rheological measurements showed higher storage and loss moduli than those of rhCol III-MA gel. HAp@rhCol III-MA did not reduce NIH-3T3 cell viability at the tested concentrations and enhanced HUVEC scratch closure and tube-network formation in vitro. Following subcutaneous implantation in rats, the microgel retained more volume than rhCol III-MA during the early and intermediate observation periods, with residual volumes of 56.58 ± 3.03 mm^3^ for HAp@rhCol III-MA and 52.80 ± 1.79 mm^3^ for rhCol III-MA at day 59; the commercial type I collagen comparator retained 123.23 ± 2.80 mm^3^. The composite caused no evident tissue injury and was associated with progressive collagen deposition and a low, declining CD68-positive response. These findings support further investigation of HAp@rhCol III-MA as an injectable dermal-filling material, while long-term persistence and clinical injection performance remain to be established.

## 1. Introduction

Dermal fillers are injected into the dermis or subcutaneous tissue to soften wrinkles, restore volume, and refine facial contours. Their use has expanded alongside the demand for procedures that provide visible correction with limited recovery time [1,2].

Facial aging reflects changes across several tissue compartments. Loss and remodeling of dermal collagen, epidermal and dermal thinning, elastic-fiber degeneration, and cumulative photodamage contribute to wrinkles and uneven pigmentation. Atrophy and redistribution of subcutaneous fat, together with changes in the underlying skeleton, further reduce structural support and produce folds or depressions [3,4]. Injectable fillers address part of this process by restoring lost volume rather than relying only on tissue tightening.

Collagen accounts for a substantial proportion of human protein and provides structural support in skin, bone, and many other tissues [5,6]. Its biological activity and extracellular-matrix function have made collagen an established scaffold material, particularly in approaches intended to support skin repair [7]. Most conventional collagen is isolated from animal tissues, which introduces batch variability and potential immunological or pathogen-related concerns. Recombinant production offers tighter control over sequence and manufacturing and avoids animal-derived contaminants. Clinical and preclinical studies have consequently examined recombinant collagen materials for burn treatment and repair of photoaged skin [8,9].

Type III collagen is a fibrillar collagen found in skin, blood vessels, visceral organs, and other extracellular matrices. It commonly forms heterotypic fibrils with type I collagen and contributes to matrix organization and mechanical signaling during tissue repair [10,11]. Recombinant type III collagen can reproduce selected human collagen sequences while offering high water solubility and low immunogenic potential. Expression and structural studies have also shown that recombinant production can yield stable collagen-like proteins when hydroxylation and assembly are adequately controlled [12]. These properties have encouraged the use of recombinant collagen platforms in skin repair, wound healing, corneal regeneration, and other tissue-engineering settings [13,14,15,16,17].

High water solubility and rapid loss from aqueous environments, however, limit unmodified rhCol III as a durable soft-tissue filler. Introducing photocrosslinkable methacryloyl groups provides a way to stabilize the collagen network after formulation. Related recombinant collagen hydrogels have supported cell adhesion and tissue repair, although the persistence and mechanical strength required for filling applications remain material-dependent [18,19,20].

For a collagen-based filler, this stabilization problem involves more than slowing bulk degradation. The formulation must pass through a needle, recover sufficient cohesion after injection, and remain localized while host tissue begins to infiltrate and remodel the implant. A densely crosslinked network may improve persistence but can reduce water exchange and cell interaction. A weak network may be biologically accessible yet lose its shape before newly deposited extracellular matrix can contribute meaningful support. The design of rhCol III-MA therefore requires a balance between processability, mechanical integrity, and exposure of collagen-derived biological cues. Photocrosslinking offers control over network formation, while a dispersed mineral phase may reinforce the network without replacing the collagen-rich interface presented to surrounding cells.

Hydroxyapatite (HAp) is the principal calcium phosphate mineral in bone and tooth enamel [21]. Synthetic HAp is formed from calcium and phosphate ions and is widely used in bone-repair materials because of its mineral composition and biological compatibility [22]. Its role is not restricted to hard tissue. Calcium hydroxyapatite microspheres are also used in injectable fillers, where a carrier gel provides early volume and the particles can induce a local fibroblast response and neocollagenesis [23,24,25,26]. Work by Nowag et al. indicated that direct contact between calcium hydroxyapatite microspheres and fibroblasts contributes to this response [27]. The combination of an initially space-filling phase and subsequent matrix remodeling makes HAp-containing formulations relevant to longer-lasting soft-tissue augmentation.

The behavior of HAp in a soft-tissue filler depends strongly on particle size, surface exposure, and its distribution within the carrier. Large agglomerates may produce an uneven injection profile, whereas very small particles can be cleared more readily by phagocytic cells. Minerals that are tightly embedded in a polymer network may contribute to mechanical reinforcement but have limited direct contact with fibroblasts. Conversely, exposed particles may increase cell-material interaction while also changing the local inflammatory response. Incorporating HAp during formation of the rhCol III-MA microgel was intended to distribute the mineral throughout the collagen phase rather than simply mixing two preformed particulate components. The resulting composite architecture was therefore evaluated with complementary imaging, elemental, spectroscopic, thermal, and mechanical methods.

Our preliminary experiments showed that rhCol III-MA microgels alone contracted rapidly and had limited mechanical stability after implantation. We therefore prepared HAp@rhCol III-MA by mineralizing a calcium phosphate phase in the presence of rhCol III-MA and then photocrosslinking the collagen phase, drawing on established in situ mineralization strategies for collagen–calcium phosphate composites [28,29]. The objective of this study was to construct an injectable hydroxyapatite-reinforced methacrylated recombinant type III collagen microgel through in situ coprecipitation and photocrosslinking and to systematically evaluate its physicochemical properties, cytocompatibility, pro-angiogenic potential, and in vivo volume retention and tissue-remodeling behavior in soft-tissue filling. Histology, collagen staining, and CD68 immunostaining were used to relate filler persistence to the surrounding tissue response.

## 2. Results and Discussion

### 2.1. Synthesis and Structure of rhCol III-MA

**Figure 1:**
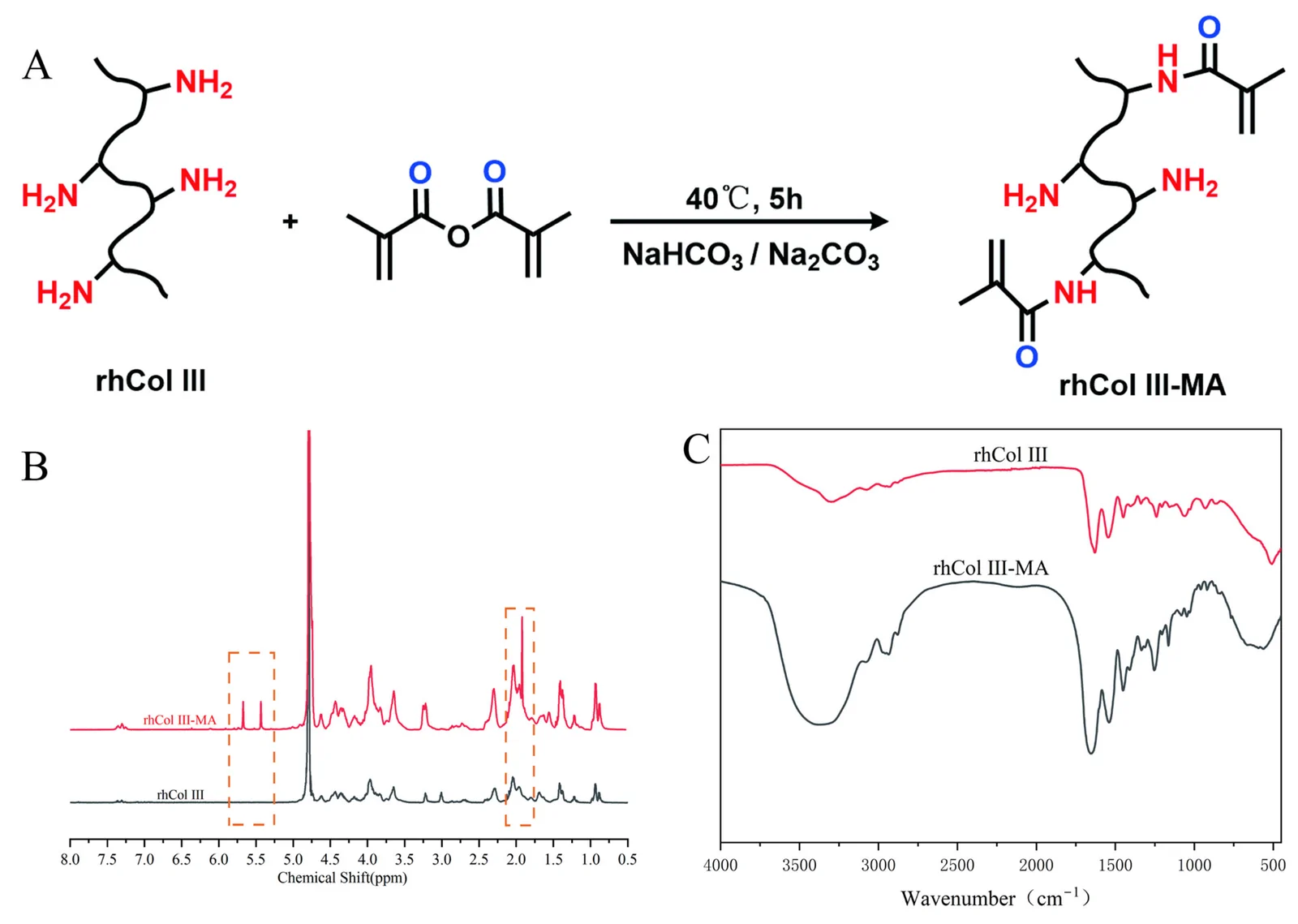
Synthesis and characterization of rhCol III-MA. (**A**) Reaction scheme and chemical structure of rhCol III-MA. (**B**) ^1^H NMR spectra of rhCol III and rhCol III-MA. (**C**) FT-IR spectra of rhCol III and rhCol III-MA.

Methacryloyl groups were introduced through acylation of amino groups on rhCol III with methacrylic anhydride, producing a photocrosslinkable rhCol III-MA derivative (Figure 1A). Compared with the spectrum of rhCol III, the 1H NMR spectrum of rhCol III-MA contained new resonances at 5.5–6.0 ppm assigned to methacryloyl vinyl protons and additional methyl-associated signals at 1.5–2.0 ppm (Figure 1B). Integration of the selected resonances gave an estimated degree of substitution of approximately 66%. The FT-IR spectrum retained the broad collagen-associated bands while showing changes in the 3000–3500 cm^−1^ region and near 1647.8 cm^−1^, together with altered features between 1000 and 1500 cm^−1^ (Figure 1C). Considered together, the NMR and FT-IR data support successful methacryloyl functionalization of rhCol III.

The spectra confirm chemical modification but do not, by themselves, establish the extent to which the collagen triple-helical conformation was retained. Structural claims beyond methacryloyl incorporation would require a conformation-sensitive measurement such as circular dichroism.

A substitution degree near 66% indicates that a substantial fraction of the available reactive sites participated in methacryloylation, while unmodified amino-containing regions remained in the collagen-derived macromolecule. Using DS (%) = (1 − P2/P1) × 100%, where P1 is the normalized integrated area of the lysine-side-chain methylene proton signal in rhCol III and P2 is the corresponding signal from residual unreacted amino groups in rhCol III-MA, the NMR integration gave an amino-group consumption of 66.4%. The newly observed vinyl-proton signal was consistent with this extent of modification within the expected integration error. This intermediate level of functionalization is relevant to the later material behavior because methacryloyl groups provide sites for radical-mediated network formation while the remaining collagen sequence continues to dominate the chemical environment of the microgel.

### 2.2. Structural and Physicochemical Properties of HAp@rhCol III-MA Microgel

#### 2.2.1. Morphology and Particle-Size Distribution

**Figure 2:**
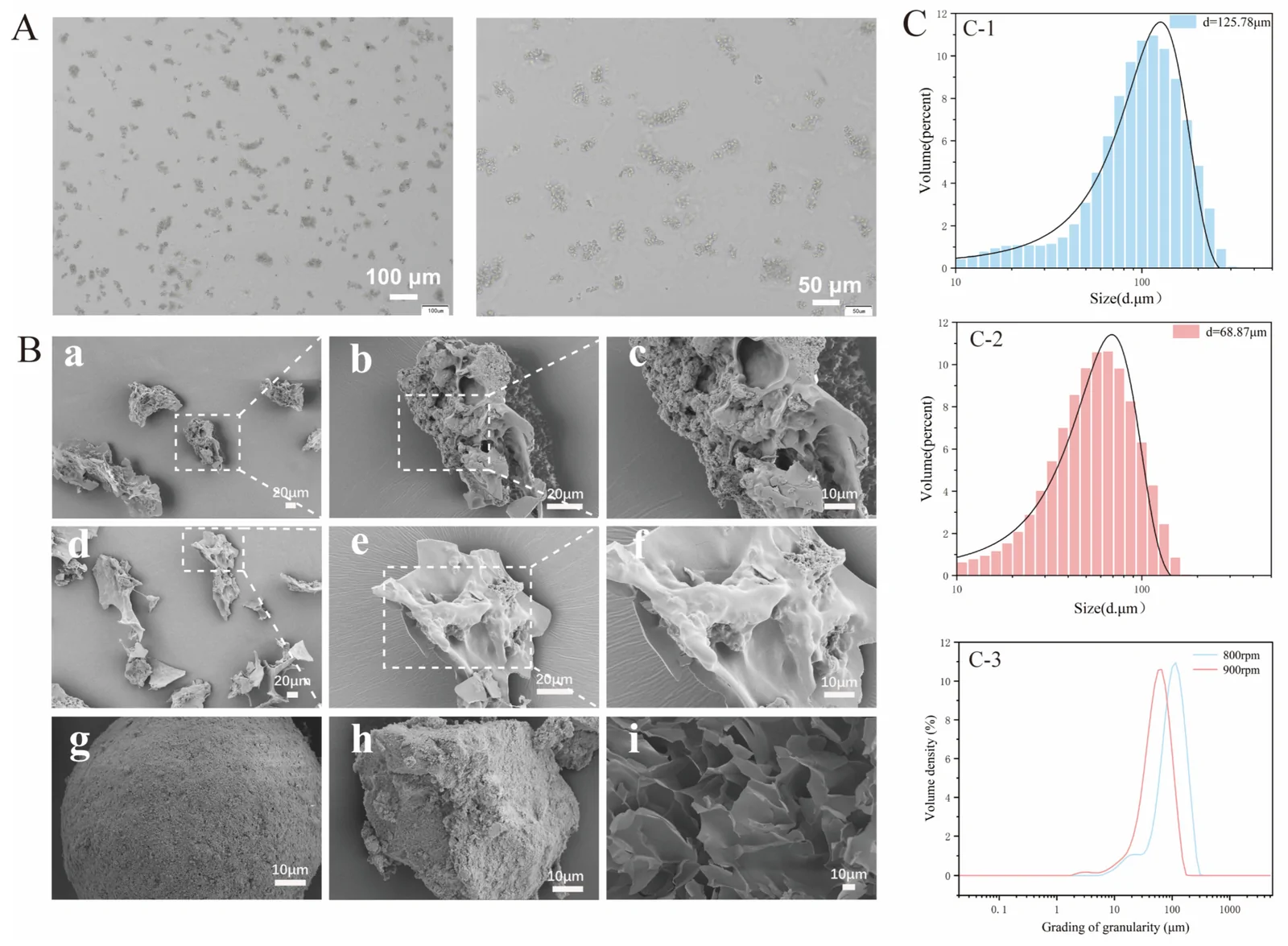
Morphology and particle-size distribution of HAp@rhCol III-MA microgels. (**A**) Optical micrographs of HAp@rhCol III-MA microgels. (**B**) SEM images of freeze-dried HAp@rhCol III-MA microgels (**a**–**f**), HAp (**g**,**h**), and rhCol III-MA gel (**i**). (**C**) Particle-size distributions obtained at different stirring **rates**: 800 rpm (**C-1**), 900 rpm (**C-2**), and direct comparison of the two conditions (**C-3**).

Optical microscopy showed dispersed, irregular HAp@rhCol III-MA particles in water (Figure 2A). After freeze-drying, SEM revealed irregular microgel fragments with a rough, porous surface (Figure 2B). Fine mineral-like deposits were distributed throughout the collagen-rich matrix and were also visible on exposed surfaces. Their appearance was comparable to that of the HAp reference, whereas the rhCol III-MA gel displayed a lamellar porous morphology. The images are consistent with a composite in which the mineral phase is associated with, rather than completely separated from, the crosslinked collagen matrix.

**Figure 3:**
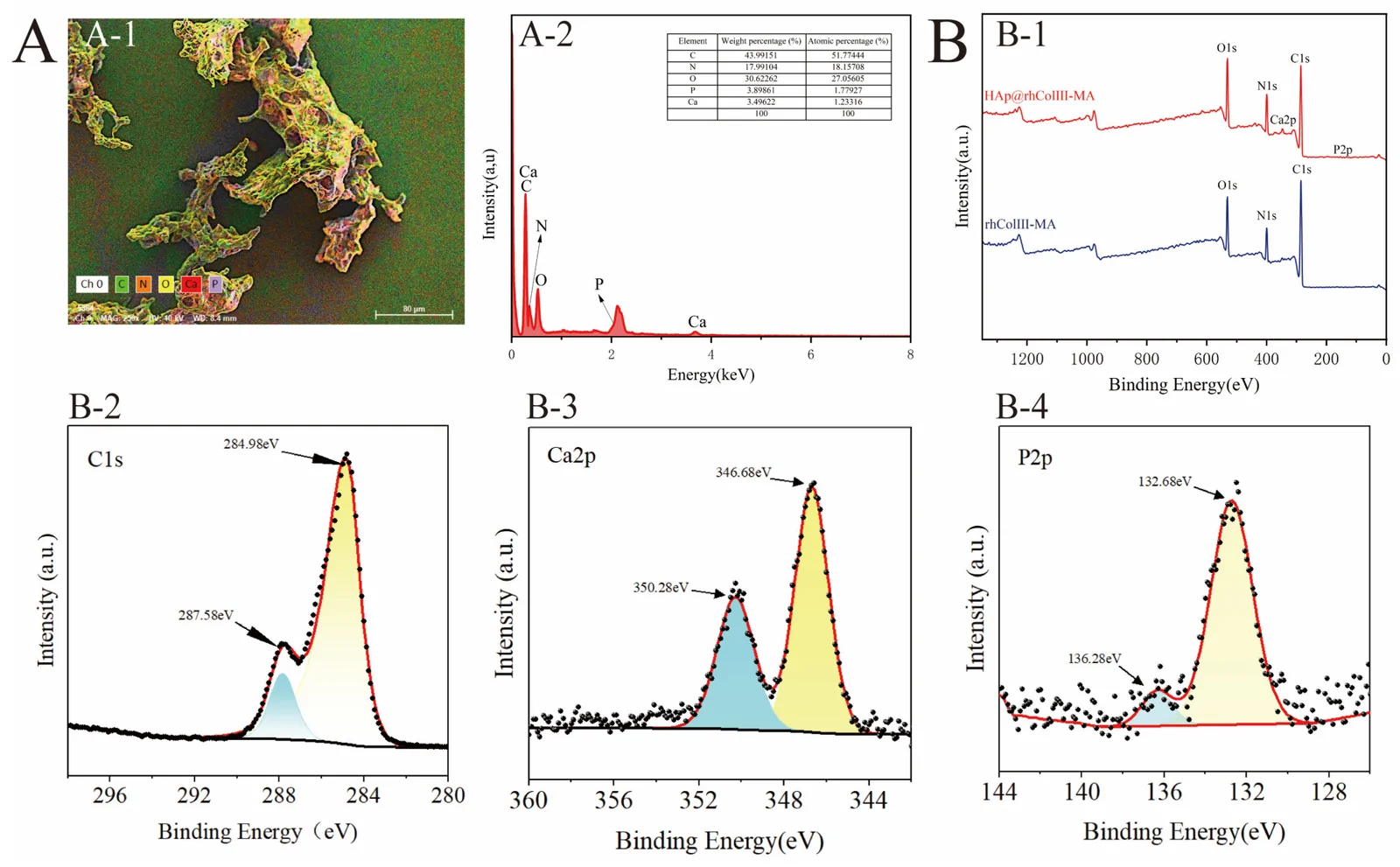
Elemental analysis of HAp@rhCol III-MA microgels. (**A**) EDS elemental mapping (**A-1**) and quantitative elemental composition (**A-2**). (**B**) XPS spectra of HAp@rhCol III-MA: survey spectrum (**B-1**), high-resolution C 1s spectrum (**B-2**), high-resolution Ca 2p spectrum (**B-3**), and high-resolution P 2p spectrum (**B-4**).

Particle size influences both injectability and the host response to particulate fillers. Particles below approximately 20 μm are more readily internalized by macrophages, whereas broad distributions containing large particles can complicate injection and tissue integration [30,31,32]. Previous implantation studies have reported favorable connective-tissue encapsulation for microspheres in the 20–80 μm range [33]. At 800 rpm, HAp@rhCol III-MA particles were distributed mainly from 50 to 150 μm, with more than 60% between 80 and 150 μm (Figure 2(C-1)). Increasing the stirring rate to 900 rpm shifted the distribution toward smaller sizes: the main range was 10–100 μm and more than 80% of particles were between 20 and 80 μm (Figure 2(C-2)). The direct comparison in Figure 2(C-3) therefore identifies 900 rpm as the more suitable preparation condition for the target particle-size range.

The shift produced by increasing the stirring speed is consistent with stronger hydrodynamic disruption during precipitation and crosslinking. At 800 rpm, growing mineral–collagen aggregates had a greater opportunity to associate before the microgel structure was fixed, producing a broader population centered at larger diameters. Stirring at 900 rpm reduced the characteristic size and narrowed the most relevant portion of the distribution. The optical and SEM images also show that the products were irregular microgels rather than geometrically uniform microspheres. Accordingly, the laser-diffraction diameters should be understood as equivalent particle sizes and not as evidence of spherical geometry. This distinction matters when considering passage through a needle, because irregular particles can orient, compress, or form transient aggregates differently from rigid spheres of the same nominal diameter. Direct extrusion-force measurements would be needed to translate the particle-size result into a quantitative claim about injectability. Within the scope of the current characterization, the 900 rpm condition provided the closest match to the intended 20–80 μm population and was carried forward as the preferred preparation condition.

#### 2.2.2. Chemical Composition and Physicochemical Properties

**Figure 4:**
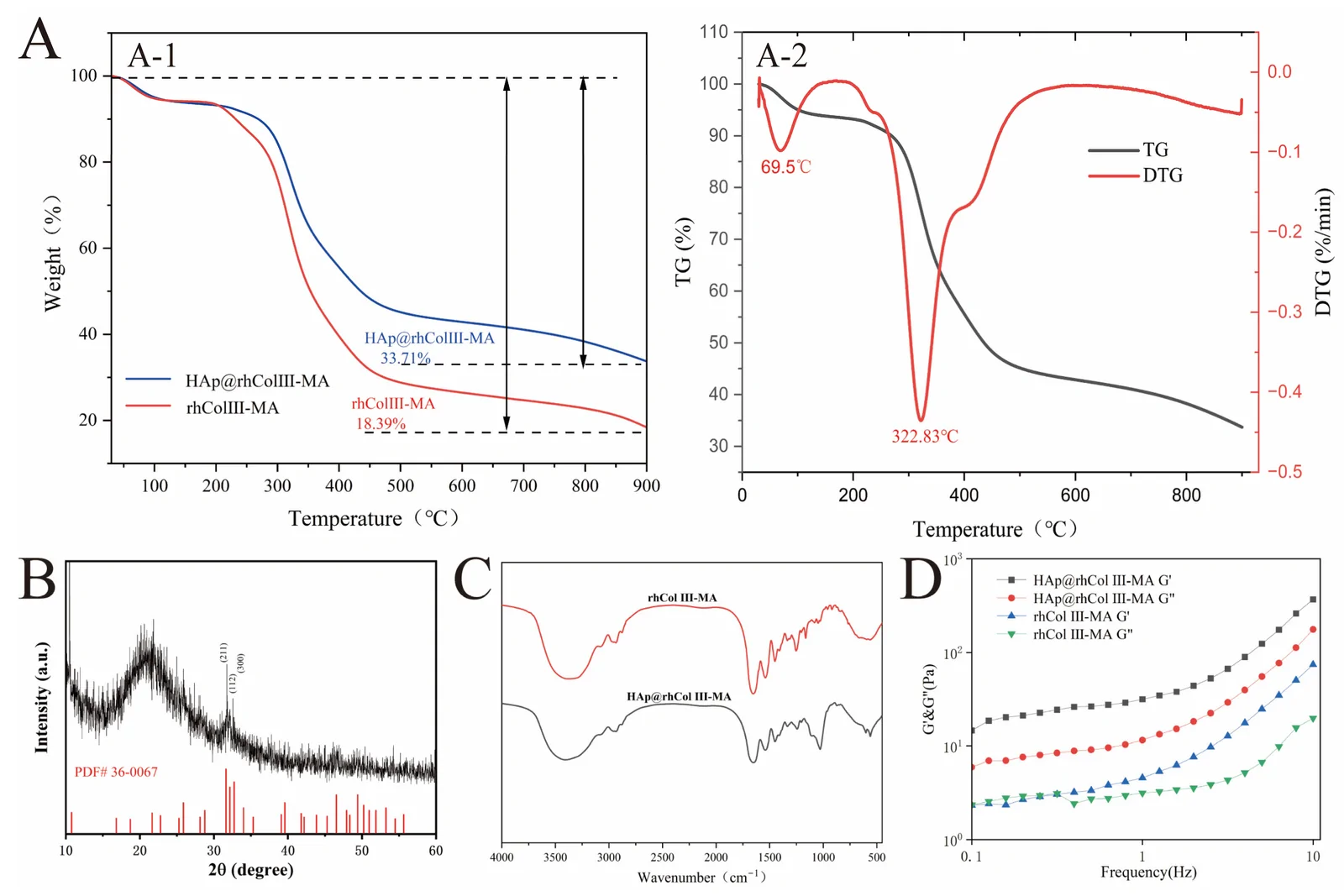
Thermal, structural, spectroscopic, and rheological analyses of the microgels. (**A**) Thermogravimetric curves (**A-1**) and derivative thermogravimetric curve of HAp@rhCol III-MA (**A-2**). (**B**) XRD pattern of HAp@rhCol III-MA and reference reflections of HAp (PDF#36-0067). (**C**) FT-IR spectra of rhCol III-MA and HAp@rhCol III-MA. (**D**) Frequency-dependent storage modulus (G′) and loss modulus (G″) of rhCol III-MA and HAp@rhCol III-MA.

EDS mapping detected Ca and P across the HAp@rhCol III-MA particles, alongside the C, N, and O signals expected from the collagen phase (Figure 3(A-1)). Quantitative EDS gave Ca and P atomic fractions of 1.23% and 1.78%, respectively, corresponding to a Ca/P ratio below stoichiometric HAp (Figure 3(A-2)). Residual phosphate-containing species from the PBS-based preparation may contribute to this deviation, so the EDS ratio should not be interpreted as the composition of a pure HAp phase. XPS provided complementary surface-sensitive evidence (Figure 3B). RhCol III-MA showed C, N, and O signals, whereas HAp@rhCol III-MA additionally displayed Ca 2p and P 2p peaks in the survey spectrum (Figure 3(B-1)). Components at 284.98 and 287.58 eV in the C 1s spectrum were assigned to hydrocarbon and carbonyl environments (Figure 3(B-2)). The Ca 2p doublet at 346.68 and 350.28 eV and the dominant P 2p component near 132.68 eV are compatible with calcium phosphate (Figure 3(B-3,B-4)) [34]. These findings support incorporation of a calcium phosphate phase into the collagen microgel.

No single compositional method is sufficient to identify a mineral phase in this type of protein-rich composite. EDS demonstrates that calcium and phosphorus occur in the same particle regions, but it samples a comparatively large interaction volume and does not by itself define chemical bonding. XPS is more surface-sensitive and identifies binding-energy environments, although peak assignments can be affected by charging, surface contamination, and overlapping components. The appearance of Ca and P in both measurements, together with their absence from the rhCol III-MA survey spectrum, provides stronger evidence than either technique alone. EDS measured Ca and P atomic fractions of 1.23% and 1.78%, respectively, while C, N, and O accounted for more than 96 at% of the analyzed region. These local elemental data are consistent with a collagen-rich composite containing a dispersed calcium phosphate phase, but they should not be converted directly into a bulk mineral mass fraction because EDS is semi-quantitative and samples a limited interaction volume. The substoichiometric Ca/P ratio of approximately 0.69 is consistent with residual phosphate associated with the PBS-based preparation and the formation of calcium-deficient, nonstoichiometric calcium phosphate. For that reason, the material is described as containing a poorly crystalline or nanocrystalline calcium phosphate phase compatible with HAp rather than as analytically pure, fully stoichiometric HAp. The approximately 15.32 wt% value obtained from TGA is therefore retained only as an apparent bulk estimate.

Thermogravimetric analysis left residual masses of 33.71% for HAp@rhCol III-MA and 18.39% for rhCol III-MA at 900 °C (Figure 4(A-1)). If the difference in residue is attributed to the mineral component, the apparent mineral content is approximately 15.32 wt%. This value is an estimate because carbonaceous residue, bound water, and inorganic salts may also remain under the measurement conditions. The derivative curve showed the principal mass-loss event near 322.8 °C, with a smaller low-temperature event near 69.5 °C that is consistent with loss of absorbed water (Figure 4(A-2)).

The XRD pattern contained a broad feature near 32.5°, overlapping the (211) reflection of the HAp reference pattern PDF#36-0067 (Figure 4B) [35]. The low intensity and broad profile indicate a poorly crystalline or nanocrystalline calcium phosphate phase within the predominantly amorphous organic matrix. This interpretation agrees with the elemental data while avoiding assignment of the composite as highly crystalline HAp.

FT-IR analysis provided further evidence of mineral incorporation. Relative to rhCol III-MA, the composite showed phosphate-associated bands near 560, 600, and 960 cm^−1^ (Figure 4C). The principal collagen-associated bands remained visible, indicating that the organic matrix persisted after coprecipitation and photocrosslinking.

Both formulations remained predominantly elastic across the tested frequency range, with G′ exceeding G″ (Figure 4D). The storage and loss moduli of HAp@rhCol III-MA were consistently higher than those of rhCol III-MA, and the separation became more pronounced at higher frequencies. HAp incorporation therefore increased the resistance of the formulation to oscillatory deformation under these test conditions.

The combined thermal, diffraction, infrared, and rheological results describe a composite in which an estimated mineral fraction changes bulk behavior without becoming the dominant phase. The higher residual mass after heating is consistent with an inorganic component, while the broad XRD signal indicates that this component lacks the long-range order of a highly crystalline HAp powder. Phosphate-associated infrared bands show that the mineral signature remains detectable within the collagen-rich material. Mechanically, the higher G′ of HAp@rhCol III-MA suggests that mineral–collagen interactions and the particulate microgel structure restrict chain motion under small-amplitude oscillation. G′ remained above G″ throughout the sweep, so neither formulation crossed into viscous-dominant behavior within the tested range. This is relevant to shape retention after placement, although oscillatory rheology does not reproduce the high shear experienced during injection. Yield stress, recovery after large strain, syringe extrusion force, needle-gauge compatibility, and measurements at physiological temperature would provide a more complete assessment. The current data support reinforcement of the rhCol III-MA network, but they do not by themselves predict clinical injection performance.

### 2.3. In Vitro Biocompatibility and Endothelial-Cell Responses

**Figure 5:**
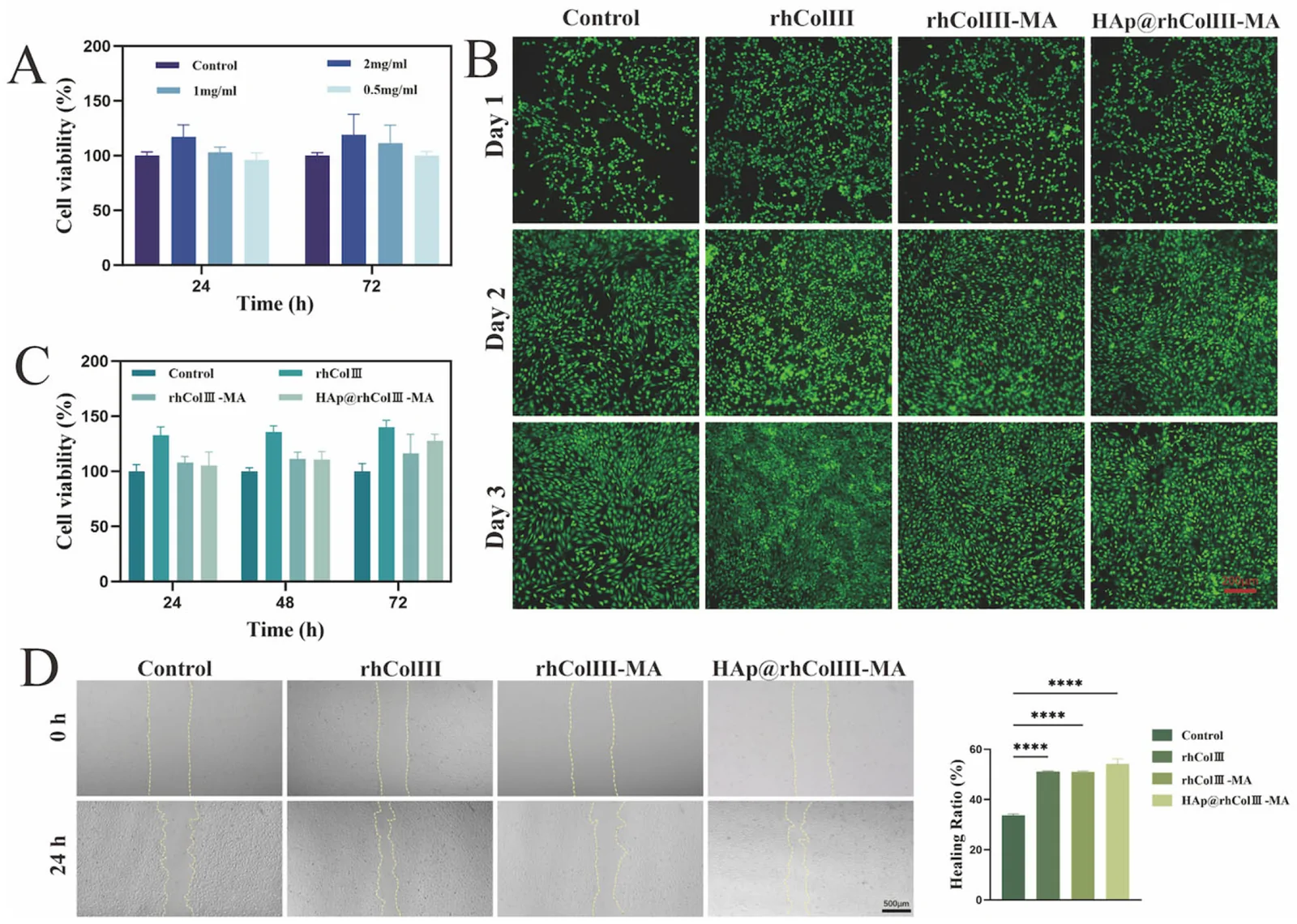
Cytocompatibility and scratch-closure analysis of the microgels. (**A**) NIH-3T3 cell viability after treatment with HAp@rhCol III-MA at 0.5, 1, and 2 mg/mL. (**B**) Live/dead fluorescence images of cells cultured with the indicated materials. Scale bar: 200 μm. (**C**) NIH-3T3 cell viability after treatment with 1 mg/mL rhCol III, rhCol III-MA, or HAp@rhCol III-MA. (**D**) Representative images and quantitative analysis of HUVEC scratch closure. Scale bar: 500 μm. Data are presented as mean ± SD (*n* = 3); **** *p* < 0.0001.

HAp@rhCol III-MA did not reduce NIH-3T3 viability at 0.5, 1, or 2 mg/mL after 24 or 72 h. Mean viability remained near or above the untreated control, although variability was greater at the higher concentrations (Figure 5A). Live/dead staining showed predominantly Calcein-AM-positive cells and few PI-positive cells in all material groups over 3 days (Figure 5B). At 1 mg/mL, viability in the rhCol III, rhCol III-MA, and HAp@rhCol III-MA groups remained above 100% of the control at each measured time point (Figure 5C). These data indicate that the three collagen-based formulations were cytocompatible within the tested concentration and exposure ranges.

The scratch assay showed greater HUVEC wound closure after 24 h in all material-treated groups than in the untreated control (Figure 5D). Healing ratios were approximately 50% for rhCol III and rhCol III-MA and about 54% for HAp@rhCol III-MA, compared with roughly 34% in the control. Each treatment differed significantly from the control, whereas no significance comparison among the three material groups was indicated. The experiment therefore supports enhanced collective cell migration or scratch closure, but it does not independently distinguish migration from proliferation.

The viability and scratch data indicate that methacryloylation and mineral incorporation preserved the favorable cellular response of the collagen-based formulations. In the CCK-8 assay, the untreated DMEM group was defined as 100%, whereas the absorbance of the treated groups reflects both viable-cell number and intracellular dehydrogenase activity. Recombinant type III collagen contains cell-interactive sequences and collagen-like structural domains that can support initial adhesion and spreading of NIH-3T3 fibroblasts and stimulate cellular metabolic activity. Soluble or partially exposed collagen signals from the microgels may therefore produce higher metabolic activity than the basal-medium control, which contains no additional adhesive matrix. Values above 100% should consequently be interpreted as increased metabolic activity relative to the reference control rather than as an abnormal viability measurement. Because scratch closure can also include contributions from migration and cell division, these assays are interpreted together as evidence of cytocompatibility and enhanced wound closure rather than as a direct measurement of proliferation alone.

**Figure 6:**
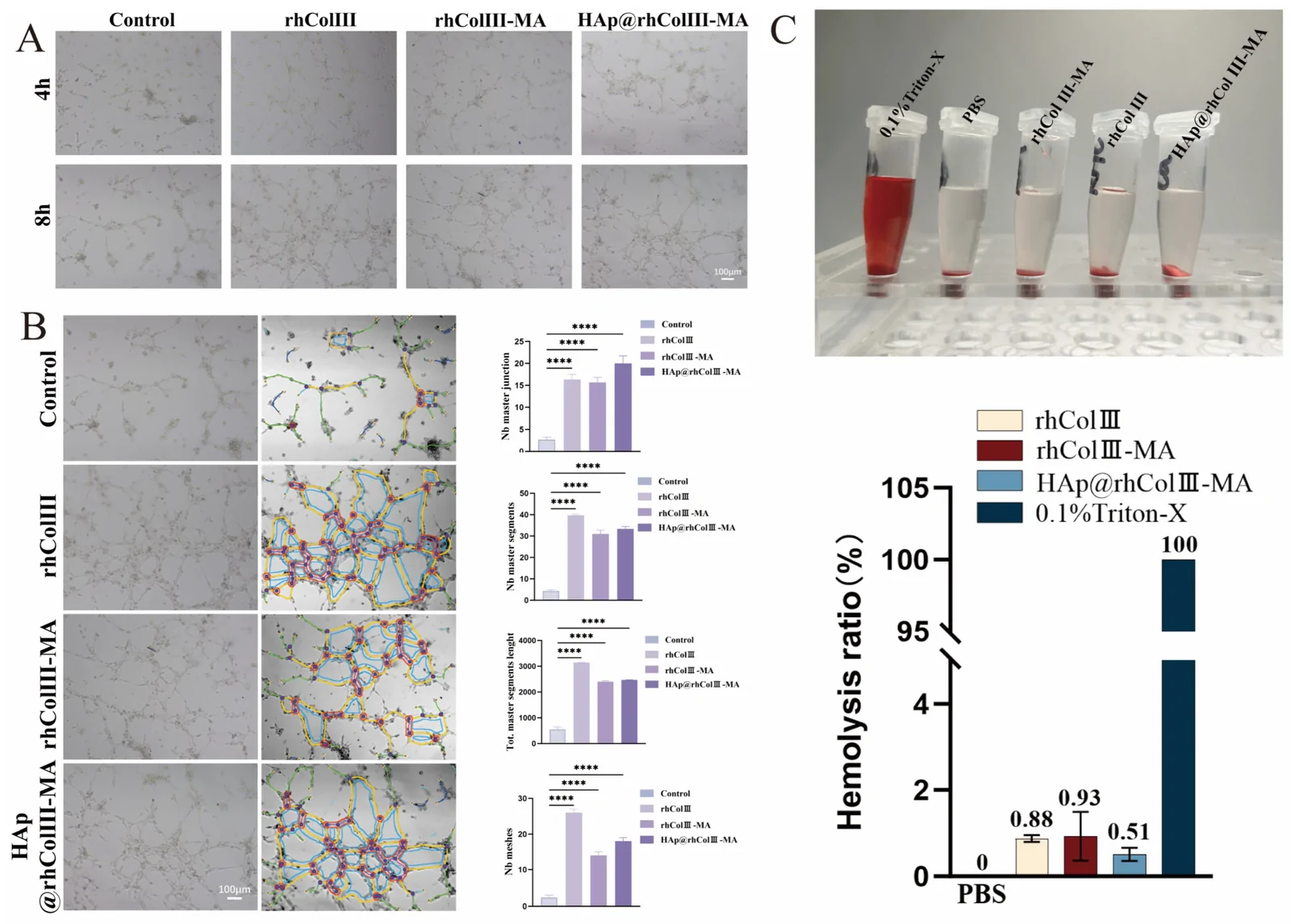
Endothelial tube formation and hemolysis analysis. (**A**) Representative optical micrographs of HUVEC tube formation at 4 and 8 h. Scale bar: 100 μm. (**B**) Quantitative analysis of master junctions, master segments, total master-segment length, and meshes at 8 h. Data are presented as mean ± SD (*n* = 3); **** *p* < 0.0001. (**C**) Representative hemolysis assay images and calculated hemolysis ratios for rhCol III, rhCol III-MA, and HAp@rhCol III-MA, with PBS and 0.1% Triton X-100 as negative and positive controls, respectively.

HUVECs formed progressively more connected tube-like networks between 4 and 8 h in the presence of each collagen-based material (Figure 6A). At 8 h, HAp@rhCol III-MA produced the highest number of master junctions, exceeding the control, rhCol III, and rhCol III-MA groups (Figure 6B). Its numbers of master segments and meshes and its total master-segment length were also markedly higher than the control values. For these three measures, however, rhCol III gave the highest means, while HAp@rhCol III-MA was close to or slightly above rhCol III-MA. The composite thus supported formation of a connected endothelial network, with a particularly strong effect on junction formation rather than uniform superiority across every network metric.

All three material groups showed low hemolysis. The measured ratios were 0.88% for rhCol III, 0.93% for rhCol III-MA, and 0.51% for HAp@rhCol III-MA, compared with 0% for PBS and 100% for the Triton X-100 control (Figure 6C). The values below 1% indicate favorable blood compatibility under the conditions of this assay.

The four tube-network descriptors capture different aspects of endothelial organization. The high junction number in the HAp@rhCol III-MA group indicates a densely connected pattern, whereas rhCol III produced the highest mean segment number, total segment length, and mesh number. Together with hemolysis below 1%, these results show that the composite supported endothelial organization and exhibited favorable blood compatibility under the tested in vitro conditions.

### 2.4. Subcutaneous Implantation of HAp@rhCol III-MA Microgel

**Figure 7:**
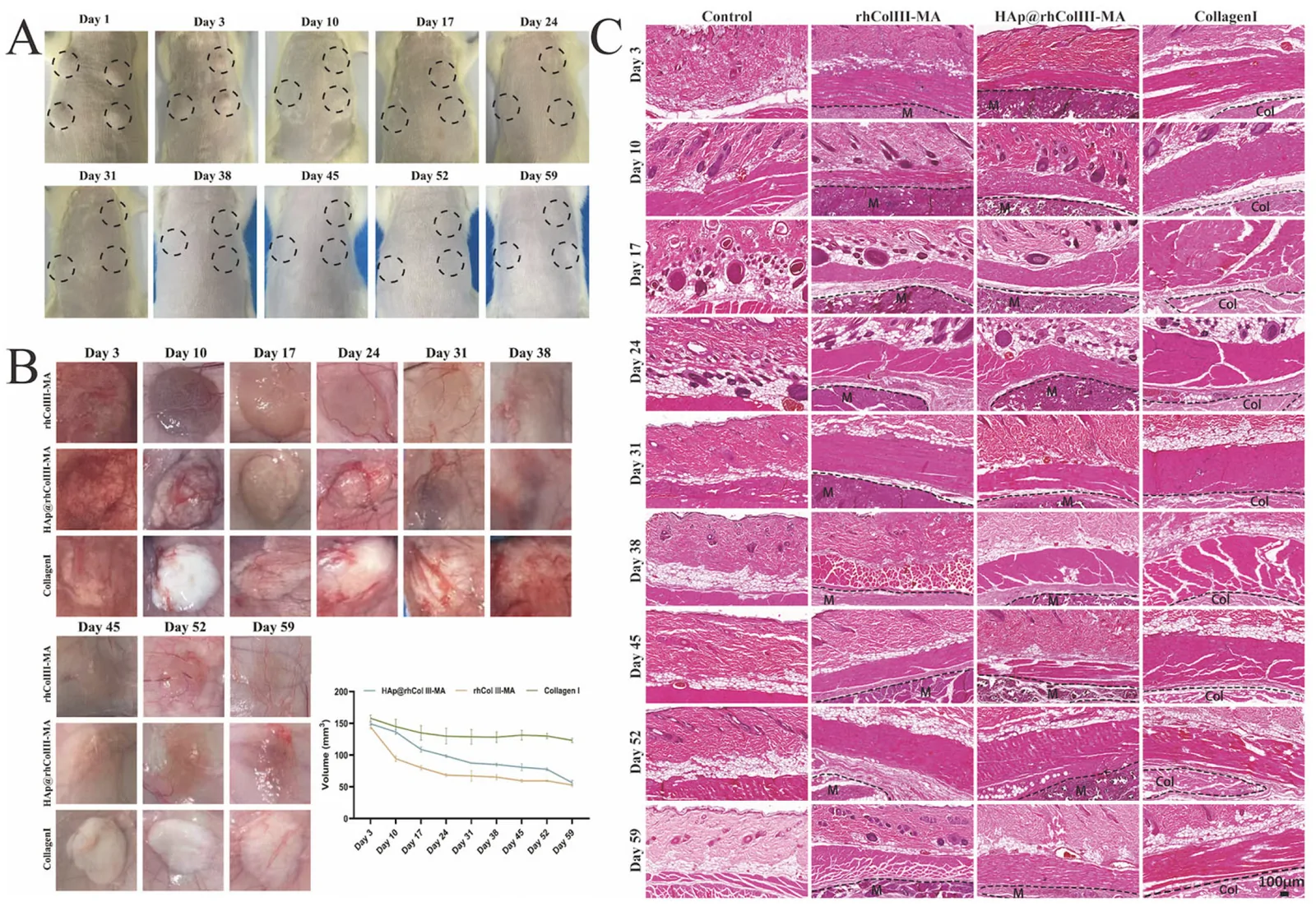
Gross and histological evaluation of the injection sites. (**A**) Dorsal injection sites from day 1 to day 59. Dashed circles indicate the injection sites: PBS control at the upper left, collagen I at the upper right, rhCol III-MA at the lower left, and HAp@rhCol III-MA at the lower right. (**B**) Gross appearance of the recovered fillers and changes in filler volume from day 3 to day 59. (**C**) Representative hematoxylin and eosin-stained sections of the injection sites. M indicates microgel and Col indicates collagen I. Scale bar: 100 μm.

Four dorsal sites were used for the PBS control, rhCol III-MA, HAp@rhCol III-MA, and collagen I formulations (Figure 7A). Across the 59-day observation period, the overlying skin remained intact without visible ulceration or infection. Gross examination of the excised sites showed progressive volume loss for both rhCol III-MA formulations, whereas collagen I retained the largest volume (Figure 7B). RhCol III-MA decreased rapidly from approximately 140 mm3 on day 3 to about 50 mm3 on day 59. HAp@rhCol III-MA declined more gradually over the early and middle time points and reached a similar final volume. The mineral-containing microgel therefore improved volume retention relative to rhCol III-MA during much of the study, although the present data do not establish persistence beyond 59 days.

Hematoxylin and eosin staining showed identifiable filler regions beneath the dermis without extensive tissue disruption or a dense inflammatory infiltrate (Figure 7C). The filler regions became smaller over time and were bordered by host tissue. These sections support local tissue tolerance during the study period, while the cellular response is assessed more specifically by CD68 staining below.

The volume curves provide a useful comparison between the two recombinant collagen formulations. HAp@rhCol III-MA separated from rhCol III-MA most clearly during the early and middle observation periods, when the composite retained a visibly larger volume. By day 59, the residual volumes were 56.58 ± 3.03 mm^3^ for HAp@rhCol III-MA and 52.80 ± 1.79 mm^3^ for rhCol III-MA, whereas collagen I retained 123.23 ± 2.80 mm^3^. This behavior is compatible with the higher rheological moduli measured in vitro, as a more constrained network would be less susceptible to rapid collapse or dispersal after hydration. Mineral incorporation therefore delayed volume loss rather than preventing it throughout the entire study. Collagen I remained the most persistent comparator under these conditions. Differences in concentration, network structure, hydration, and formulation history may all contribute to that comparison, so the curves should not be interpreted solely as a difference between collagen types or as evidence of superiority over the benchmark filler.

**Figure 8:**
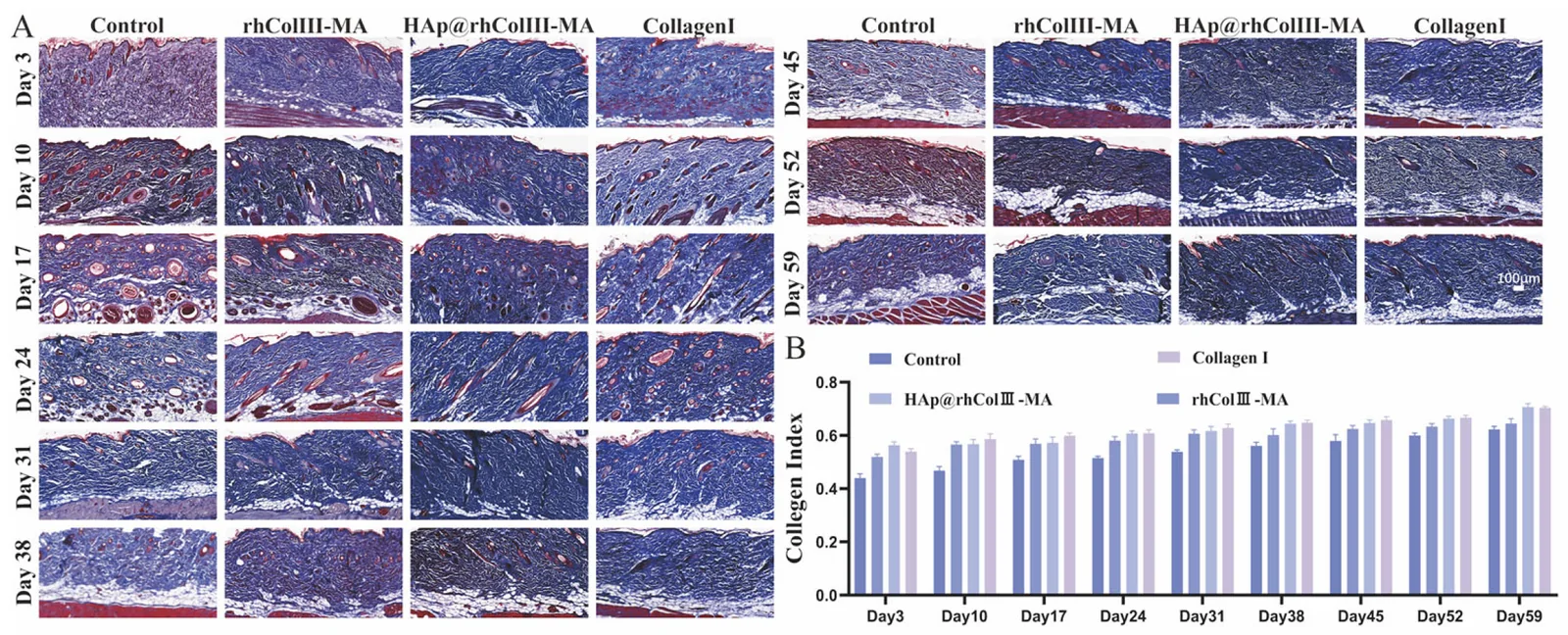
Masson’s trichrome staining and collagen-index analysis. (**A**) Representative Masson’s trichrome-stained sections of the injection sites from day 3 to day 59. Scale bar: 100 μm. (**B**) Collagen index quantified from the Masson-stained sections.

Masson’s trichrome staining showed collagen-rich tissue at and around the injection sites in all groups, with the collagen index increasing over time (Figure 8A,B). The HAp@rhCol III-MA group generally showed a higher collagen index than the untreated control and values comparable to the other filler groups. Because no pairwise significance markers are provided in Figure 8B, the data support an association with collagen deposition but not a claim of statistical superiority over rhCol III-MA or collagen I. The histology therefore suggests that the composite combined physical filling with progressive integration into a collagen-rich matrix.

Masson’s trichrome and polarized Sirius Red imaging describe complementary features of matrix remodeling. The increase in collagen-rich area, followed by a gradual rise in the collagen I/III ratio after days 24–31, is consistent with continued matrix deposition and maturation around the filler sites. The untreated control also changed over time, indicating that injection-associated repair contributed to the response, while the generally higher collagen indices in the filler groups are compatible with additional matrix formation around the implants.

**Figure 9:**
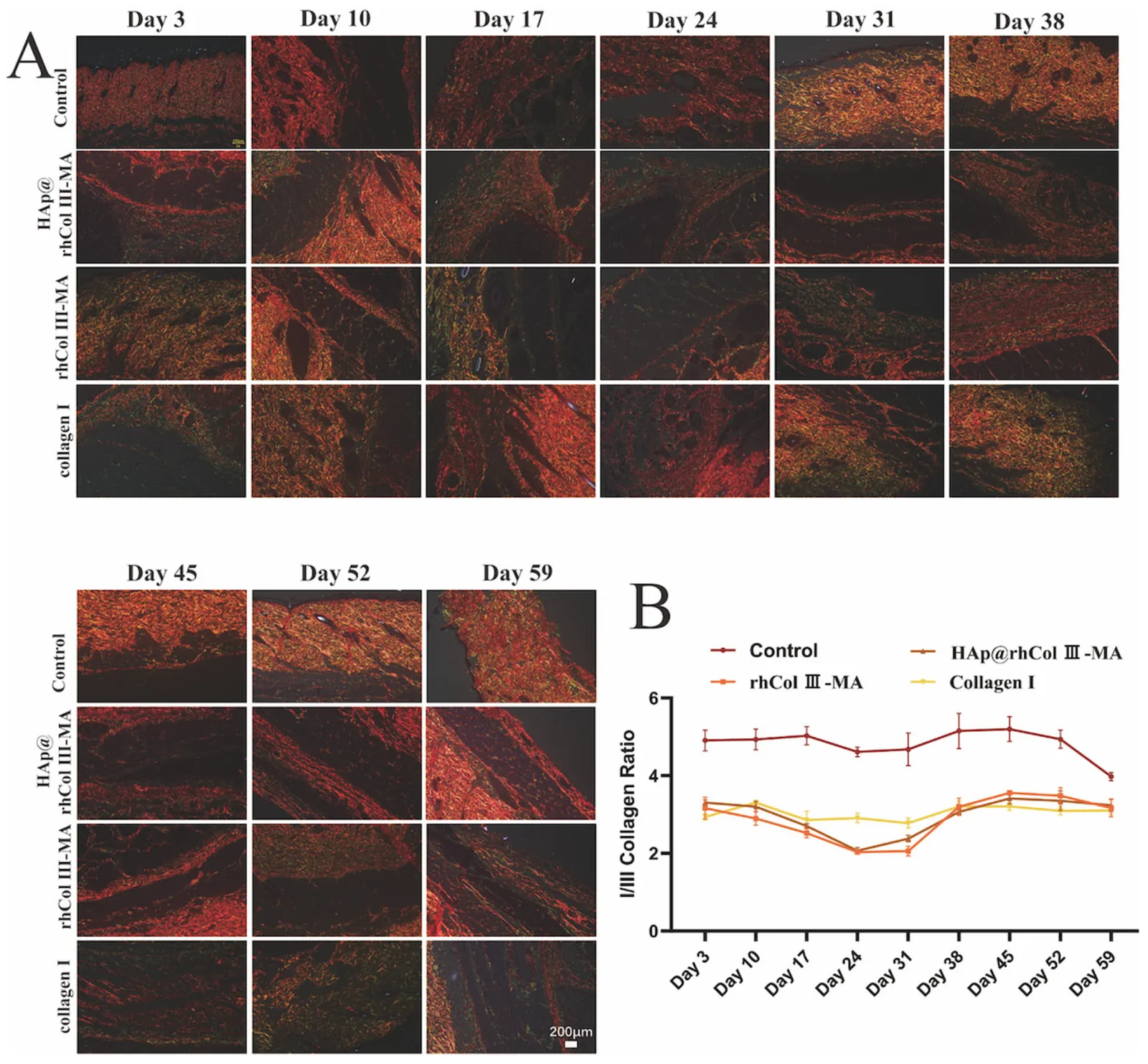
Sirius Red staining and collagen I/III ratio analysis. (**A**) Representative polarized-light micrographs of Sirius Red-stained sections from day 3 to day 59. Scale bar: 200 μm. (**B**) Quantitative analysis of the collagen I/III ratio.

Sirius Red staining under polarized light revealed both type I and type III collagen-associated birefringence around the injection sites (Figure 9A) [36]. The control maintained the highest collagen I/III ratio throughout most of the study, generally near 4–5. In the three filler groups, the ratio decreased during the early-to-middle phase, reaching its lowest values around days 24–31, and then rose toward approximately 3 by day 59 (Figure 9B). This pattern is consistent with the established sequence of tissue repair: during the early repair and granulation-tissue stages, activated fibroblasts produce relatively flexible type III collagen as a provisional matrix, whereas during the later remodeling phase, this provisional matrix is progressively reorganized and replaced by mechanically stronger type I collagen. The data describe matrix remodeling over 59 days but do not establish equivalence to uninjured young skin or long-term tissue maturation.

The temporal shift from a relatively larger type III collagen contribution toward a higher type I/III ratio occurred alongside continued collagen accumulation in the Masson analysis. This parallel pattern supports progressive organization of the newly formed matrix during the 59-day observation period.

**Figure 10:**
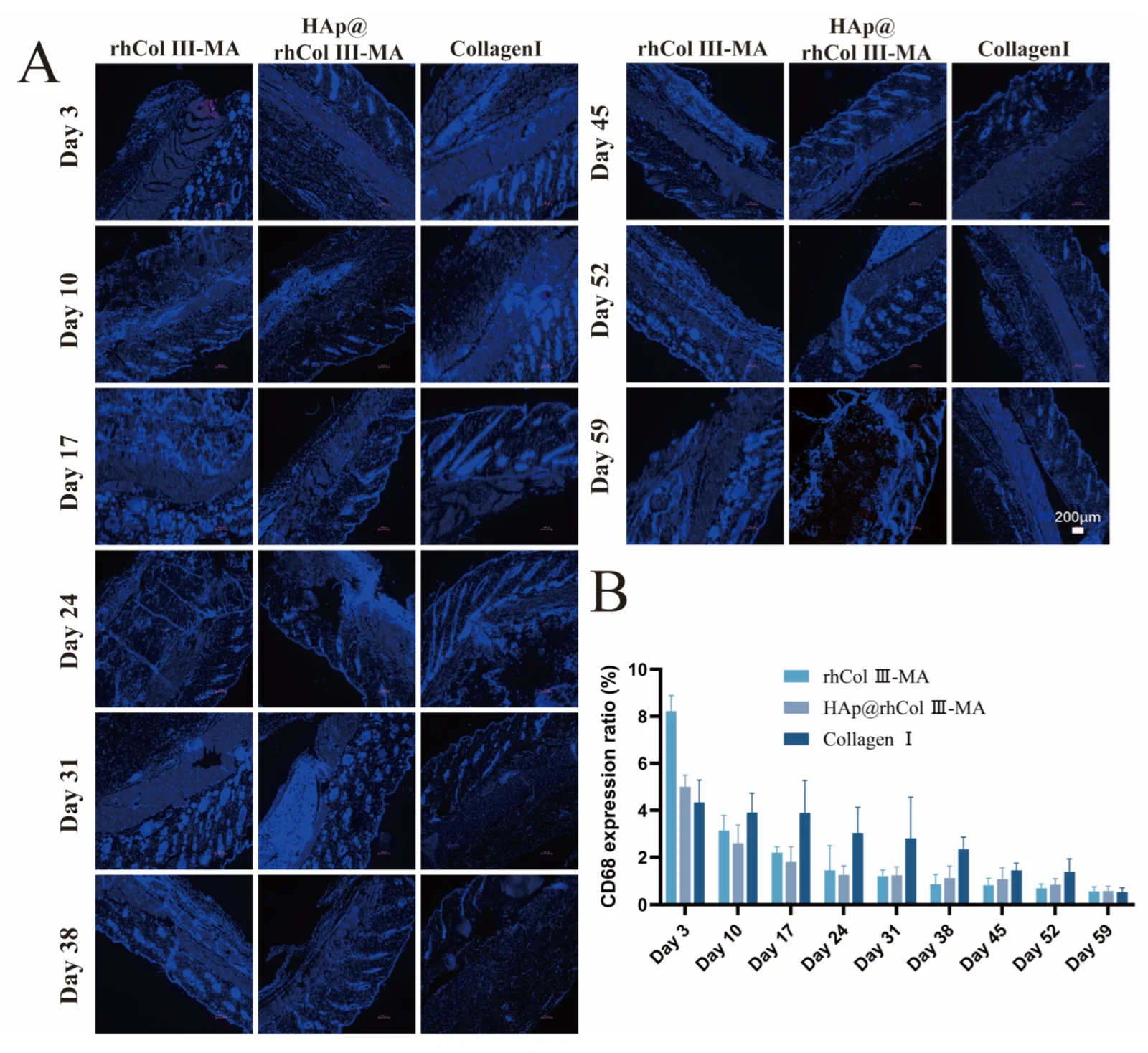
CD68 immunofluorescence staining and quantitative analysis. (**A**) Representative fluorescence images of CD68 staining at the injection sites from day 3 to day 59. Scale bar: 200 μm. (**B**) Quantitative analysis of the CD68-positive area in the rhCol III-MA, HAp@rhCol III-MA, and collagen I groups.

CD68 immunofluorescence was most apparent at the early time points and declined thereafter in all filler groups (Figure 10A). Quantification showed that rhCol III-MA had the highest mean CD68-positive area on day 3, at approximately 8%, whereas HAp@rhCol III-MA and collagen I were near 5% and 4%, respectively (Figure 10B). Values decreased to around or below 1% by day 59. The low and progressively declining signal indicates that none of the tested fillers produced a sustained macrophage-rich response during the observation period.

The declining CD68 profile is consistent with an early macrophage response that subsided as the implantation sites stabilized. HAp@rhCol III-MA did not show a greater early CD68-positive area than rhCol III-MA, indicating that incorporation of the calcium phosphate phase did not intensify the measured macrophage response.

The results should be interpreted within the scope of the present experiments. CCK-8 and scratch assays do not independently resolve proliferation from migration, and the tube-formation assay represents short-term endothelial organization rather than mature angiogenesis. Caliper-based filler volumes are estimates of irregular deposits, while Masson, Sirius Red, and CD68 image analyses remain semiquantitative and depend on section selection and thresholding. The 59-day implantation period supports comparison of early and intermediate remodeling but not claims of year-long persistence. Longer implantation, collagen-specific biochemical or immunohistochemical measurements, degradation-product analysis, and standardized extrusion testing will be needed to relate the observed tissue response to long-term filling performance.

## 3. Conclusions

RhCol III was successfully functionalized with methacryloyl groups and combined with an in situ-formed poorly crystalline calcium phosphate phase compatible with HAp to produce HAp@rhCol III-MA microgels. Preparation at 900 rpm yielded a particle population in which more than 80% of particles were 20–80 μm. Elemental mapping, XPS, XRD, FT-IR, and thermogravimetric data collectively supported mineral incorporation, while rheological measurements showed higher moduli than those of rhCol III-MA gel. The composite was cytocompatible at 0.5–2 mg/mL, enhanced HUVEC scratch closure, supported endothelial tube-network formation, and caused less than 1% hemolysis. Masson’s trichrome and Sirius Red staining demonstrated progressive collagen deposition and dynamic matrix remodeling across all filler-treated groups, including rhCol III-MA, HAp@rhCol III-MA, and collagen I, over the 59-day period. In rats, HAp@rhCol III-MA retained more volume than rhCol III-MA during much of the 59-day study and was surrounded by collagen-rich tissue with a low, declining CD68-positive response. These results support further investigation of HAp@rhCol III-MA as a candidate for injectable soft-tissue filling, but longer implantation studies, direct injectability measurements, degradation analysis, and appropriately powered comparisons with clinical benchmark fillers are still needed before durability or clinical performance can be established.

The study also defines several practical boundaries for subsequent development. Particle size was controlled through stirring rate, but batch-to-batch reproducibility and the fraction of particles outside the target range were not assessed. Mineral content was estimated indirectly from thermogravimetric residue at approximately 15.32 wt% and should be confirmed with a calcium- or phosphorus-specific quantitative method. Rheological reinforcement was demonstrated under small oscillatory strain, while recovery after needle extrusion, yield stress, needle-gauge compatibility, and standardized syringe-extrusion force remain unknown. The animal study established a 59-day local response and comparative volume trend, not long-term filler duration. Resolving these points will clarify whether the favorable biological response can be combined with persistence and handling characteristics suitable for a clinically useful formulation.

## 4. Materials and Methods

### 4.1. Experimental Materials

RhCol III was obtained from Guangzhou Peptide Source Biotechnology Co., Ltd. (Guangzhou, China). Methacrylic anhydride was purchased from Sigma-Aldrich. Calcium nitrate tetrahydrate and diammonium hydrogen phosphate were obtained from Shanghai Aladdin Biochemical Technology Co., Ltd. (St. Louis, MO, USA) and Shanghai Macklin Biochemical Technology Co., Ltd. (Shanghai, China), respectively. Matrix-Gel and Cell Counting Kit-8 (CCK-8) were purchased from Beyotime Biotechnology Co., Ltd. (Shanghai, China). Dulbecco’s modified Eagle’s medium (DMEM), fetal bovine serum (FBS), and trypsin were obtained from Gibco (Grand Island, NY, USA). Hematoxylin and eosin staining solution was obtained from Shanghai Yuanye Bio-Technology Co., Ltd. (Shanghai, China), Masson staining solution from Beijing Solarbio Science & Technology Co., Ltd. (Beijing, China), and Sirius Red staining solution from Fuzhou Feijing Biotechnology Co., Ltd. (Fuzhou, China). Anti-CD68 antibody and goat anti-rabbit IgG were obtained from Abcam (Cambridge, UK). The NIH-3T3 cell line was originally obtained from the Cell Experiment Center of Sun Yat-sen University (Guangzhou, China). All chemicals were of analytical grade and were used without further purification.

The amino acid sequence of rhCol III was EAGIPGVPGAKGEDGKDGSPGEPGANGLPGAAGERGAPGFRGPAGPNGIPGEKGPAGERGAPGPAGPRGAAGEPGRDGVPGGPGMRGMPGSPGGPGSDGKPGPPGSQGESGRPGPPGPSGPRGQPGVMGFPGPKGNDGAPGKNGERGGPGGPGPQGPPGKNGETGPQGPPGPTGPGGDKGDTGPPGPQGLQGLPGTGGPPGENGKPGEPGPKGDAGAPGAPGGKGDAGAPGERGPPGLAGAPGLRGGAGPPGPEGGKGAAGPPGPPGAAGTPGLQGMPGERGGLGSPGPKGDKGEPGGPGADGVPGKDGPRGPTGPIGPPGPAGQPGDKGEGGAPGLPGIAGPRGSPGERGETGPPGPAGFPGAPGQNGEPGGKGERGAPGEKGEGGPPGVAGPPGGSGPAGPPGPQGVKGERGSPGGPGAAGFPGARGLPGPPGSNGNPGPPGPSGSPGKDGPPGPAGNTGAPGSPGVSGPKGDAGQPGEKGSPGAQGPPGAPGPLGIAGITGARGLAGPPGMPGPRGSPGPQGVKGESGKPGANGLSGERGPPGPQGLPGLAGTAGEPGRDGNPGSDGLPGRDGSPGGKGDRGENGSPGAPGAPGHPGPPGPVGPAGKSGDRGESGPAGPAGAPGPAGSRGAPGPQGPRGDKGETGERGAAGIKGHRGFPGNPGAPGSPGPAGQQGAIGSPGPAGPRGPVGPSGPPGKDGTSGHPGPIGPPGPRGNRGERGSEGSPGHPGQPGPPGPPGAPGP.

The reported sequence contains 745 amino acids and corresponds to a continuous native fragment of the human type III collagen alpha-1 chain (COL3A1), spanning residues 451–1195 in the reference sequence. It consists of continuous Gly-X-Y collagen-like repeats and was produced by recombinant secretion in Pichia pastoris.

### 4.2. Synthesis and Characterization of rhCol III-MA

#### 4.2.1. Synthesis of rhCol III-MA

RhCol III (1 g) was dissolved in 10 mL of 0.1 M Na_2_CO_3_–NaHCO_3_ buffer adjusted to pH 9.0 under continuous stirring at room temperature. The solution was transferred to a 40 °C water bath, and 0.2 mL of methacrylic anhydride was added dropwise at 0.5 mL/min while stirring. The reaction was continued at 40 °C for 5 h. The reaction mixture was then transferred to a dialysis membrane with a molecular-weight cutoff of 1 kDa and dialyzed against ultrapure water for 3 days. The dialyzed product was freeze-dried, designated rhCol III-MA, and stored protected from light.

#### 4.2.2. Chemical Characterization of rhCol III-MA

Freeze-dried rhCol III and rhCol III-MA were separately mixed with potassium bromide at a sample-to-KBr mass ratio of 1:100, thoroughly ground, and pressed into pellets. FT-IR spectra were recorded using a Nicolet 5700 spectrometer (Thermo Fisher Scientific, Waltham, MA, USA) from 500 to 4000 cm^−1^ at a resolution of 4 cm^−1^ with 32 scans.

For proton nuclear magnetic resonance (1H NMR) analysis, appropriate amounts of rhCol III and rhCol III-MA were separately dissolved in D2O and transferred to NMR tubes after complete dissolution. Spectra were acquired using a 500 MHz AVANCE III 500 spectrometer (Bruker, Karlsruhe, Germany) and processed with MestReNova (Mestrelab Research, Santiago de Compostela, Spain). The degree of substitution was calculated as DS (%) = (1 − P_2_/P_1_) × 100%.

$$DS\left(\%\right)=\left(1-\frac{P_{2}}{P_{1}}\right)\times 100\%$$

Here, P1 is the normalized integrated area of the lysine-side-chain methylene proton signal in unmodified rhCol III, and P2 is the corresponding normalized integrated area of residual unreacted amino groups in rhCol III-MA. The selected signal assignments and the calculation logic were used to obtain the reported degree of substitution of approximately 66.4%.

### 4.3. Preparation of HAp@rhCol III-MA Microgel

RhCol III-MA (180 mg) was dissolved in 1.8 mL of PBS to obtain a 100 mg/mL solution. Calcium nitrate tetrahydrate (70.85 mg) was added and dissolved with stirring. A solution containing 21.46 mg of diammonium hydrogen phosphate was then introduced under magnetic stirring, giving a nominal precursor Ca/P molar ratio of 1.667 [37]. This starting ratio was selected for the precipitation reaction but does not imply formation of a fully crystalline or stoichiometric HAp phase. After phosphate addition, 650 μL of 0.5 M NaOH was added. The reaction mixture reached a measured pH of 10.2 ± 0.8 and was stirred at 900 rpm and 37 °C for 2 h to promote formation of a poorly crystalline calcium phosphate phase compatible with HAp in the presence of the rhCol III-MA chains. PBS (1.8 mL) containing 0.5% (*w*/*v*) I2959 photoinitiator was then added, and the suspension was stirred under 365 nm ultraviolet irradiation at a measured sample-surface irradiance of 15 mW/cm^2^ for 15 min to crosslink rhCol III-MA. The resulting HAp@rhCol III-MA microgel was collected, washed with ultrapure water and 75% ethanol, and freeze-dried.

### 4.4. Structural and Physicochemical Characterization of HAp@rhCol III-MA Microgel

#### 4.4.1. Morphology and Particle-Size Analysis

HAp@rhCol III-MA microgel was dispersed in ultrapure water, placed on a microscope slide, and imaged by optical microscopy. The diameters of 30–50 particles were measured from the optical micrographs using ImageJ (version 1.53) to obtain a preliminary size distribution. Particle-size distributions were also measured with a Mastersizer 3000 laser diffraction particle-size analyzer (Malvern Instruments, Malvern, UK).

#### 4.4.2. Scanning Electron Microscopy and Elemental Analysis

Freeze-dried HAp@rhCol III-MA microgels were spread on conductive adhesive attached to specimen stubs, sputter-coated with gold, and examined by scanning electron microscopy at an accelerating voltage of 5 kV. Elemental composition and spatial distribution were analyzed by energy-dispersive X-ray spectroscopy.

#### 4.4.3. X-Ray Photoelectron Spectroscopy

The elemental composition and chemical states of freeze-dried HAp@rhCol III-MA were examined by X-ray photoelectron spectroscopy using a K-Alpha+ instrument (Thermo Scientific, East Grinstead, UK) with a monochromatic Al Kα source operated at 90 W. Measurements were performed under a chamber pressure of no more than 10^−6^ mbar. Survey spectra and high-resolution spectra of C 1s, N 1s, O 1s, P 2p, and Ca 2p were collected at a pass energy of 40 eV.

#### 4.4.4. Thermogravimetric Analysis

Thermogravimetric analysis was performed with a TGA/DSC 3+ analyzer (Mettler Toledo, Greifensee, Switzerland). Samples were heated from 30 to 900 °C in air at 10 °C/min, And residual mass and derivative mass-loss profiles were recorded.

#### 4.4.5. X-Ray Diffraction Analysis

X-ray diffraction patterns were collected using a D8 Advance diffractometer (Bruker, Karlsruhe, Germany) with Cu Kα radiation generated at 40 kV. Data were recorded over a 2θ range of 10–60° at a scanning rate of 5°/min.

#### 4.4.6. FT-IR Analysis

FT-IR spectra of freeze-dried HAp@rhCol III-MA were acquired using the procedure described in Section 4.2.2.

#### 4.4.7. Rheological Analysis

The dynamic rheological behavior of HAp@rhCol III-MA microgel and rhCol III-MA gel was measured with a Kinexus Pro rotational rheometer (Malvern Instruments, UK). Storage modulus (G′) and loss modulus (G″) were recorded during frequency sweeps from 0.1 to 10 Hz at a constant strain of 1%.

### 4.5. In Vitro Biocompatibility of HAp@rhCol III-MA Microgel

#### 4.5.1. Hemolysis Assay

Fresh anticoagulated rabbit blood was centrifuged at 2000 rpm for 5 min. After removal of the supernatant, the erythrocyte pellet was diluted with PBS to obtain a 10% red blood cell suspension. Freeze-dried rhCol III, rhCol III-MA, or HAp@rhCol III-MA was prepared at 10 mg/mL and mixed 1:1 by volume with the 10% red blood cell suspension, giving a final material concentration of 5 mg/mL. Erythrocytes incubated with PBS and 0.1% Triton X-100 served as negative and positive controls, respectively. Samples were gently agitated at 90 rpm and 37 °C for 1 h and then centrifuged at 4000 rpm for 5 min. Aliquots of the supernatant (200 μL) were transferred to a 96-well plate, and absorbance was measured at 540 nm. Hemolysis was calculated using the equation below.

$$Hemolysis(\%)=\frac{{OD}_{sample}-{OD}_{negative}}{{OD}_{positive}-{OD}_{negative}}\times 100\%$$

#### 4.5.2. Cytocompatibility Assays

HAp@rhCol III-MA culture suspensions were prepared by exposing 20 mg of freeze-dried microgel to ultraviolet light for 24 h and dispersing it in 10 mL of DMEM to obtain 2 mg/mL. The suspension was serially diluted with DMEM to 1 and 0.5 mg/mL. NIH-3T3 mouse embryonic fibroblasts were seeded in 96-well plates at 3 × 10^3^ cells per well and cultured for 24 h. The medium was then replaced with 200 μL of the respective microgel suspension, with DMEM serving as the control. After 24, 48, or 72 h, 20 μL of CCK-8 reagent was added to each well and incubated for 1 h. Absorbance was measured at 450 nm using a Model 550 microplate reader (Bio-Rad, Hercules, CA, USA), and cell viability was calculated using the equation below.

$$Cell\;viability(\%)=\frac{{OD}_{test}}{{OD}_{control}}\times 100\%$$

A concentration of 1 mg/mL was used to compare rhCol III, rhCol III-MA, and HAp@rhCol III-MA. NIH-3T3 cells were cultured with each material for 24, 48, or 72 h. CCK-8 reagent was added at one tenth of the culture volume and incubated for 1 h. Absorbance was measured at 450 nm, and viability was calculated relative to the DMEM control.

Live/dead staining was performed in parallel. NIH-3T3 cells were seeded in 96-well plates at 3 × 10^3^ cells per well and cultured for 24 h. RhCol III, rhCol III-MA, and HAp@rhCol III-MA were prepared in DMEM at 1 mg/mL and added to the cells for 24, 48, or 72 h. After three washes with PBS, cells were stained in the dark with 200 μL of 0.05% Calcein-AM for 30 min and 200 μL of 0.05% PI for 10 min. The staining solution was replaced with PBS, and fluorescence images were acquired using an ECLIPSE Ti2-A inverted microscope (Nikon, Tokyo, Japan).

#### 4.5.3. Cell Scratch Assay

RhCol III, rhCol III-MA, and HAp@rhCol III-MA were each exposed to ultraviolet light for 24 h and dispersed in DMEM at 1 mg/mL. HUVECs were seeded in six-well plates at 5 × 10^5^ cells per well and cultured at 37 °C and 5% CO_2_. When the monolayer reached at least 90% confluence, two perpendicular scratches were made with a 200 μL pipette tip. Detached cells were removed by washing with PBS, and 1 mL of the corresponding material suspension was added to each well. Untreated cells served as the control. Scratch areas were imaged at 0 and 24 h, and the healing ratio was quantified from the images.

#### 4.5.4. Endothelial Tube-Formation Assay

Matrix-Gel (20 μL) was added to the center of each well of a 24-well plate, spread evenly without introducing bubbles, and polymerized at 37 °C for 30 min. HUVECs were seeded onto the gel at 1 × 10^5^ cells per well. RhCol III, rhCol III-MA, or HAp@rhCol III-MA prepared in DMEM at 1 mg/mL was added to the corresponding wells, with untreated cells serving as the control. Network formation was imaged by optical microscopy at 4 and 8 h. Master junctions, master segments, meshes, and total master-segment length were quantified at 8 h.

### 4.6. Animal Experiments

Animal procedures were approved by the Institutional Animal Care and Use Committee of Jinan University (approval no. 20240628-02) and were conducted in accordance with applicable guidelines for the care and use of laboratory animals. Twenty-seven female Sprague–Dawley rats, 6–8 weeks old and weighing 180–200 g, were acclimated for 5–7 days with free access to food and water. Freeze-dried HAp@rhCol III-MA and rhCol III-MA were sterilized by ultraviolet exposure overnight, dispersed in sterile PBS at 15 mg/mL, exposed to ultraviolet light for a further 12 h, and stored at 4 °C until use. Rats were anesthetized with inhaled isoflurane, and the dorsal hair was removed. Four circular injection areas were marked on the dorsum. Each rat received 400 μL at each site: PBS at the upper-left site, collagen I at the upper-right site, rhCol III-MA at the lower-left site, and HAp@rhCol III-MA at the lower-right site. Animals were monitored for local redness, swelling, and discharge during the first 3 days. Three rats were randomly selected at each time point and euthanized by anesthetic overdose followed by cervical dislocation on days 3, 10, 17, 24, 31, 38, 45, 52, and 59. Injection sites were photographed and collected with full-thickness skin. Filler length, width, and height were measured with digital calipers, filler volume was estimated using the equation below, and tissues were fixed in 4% paraformaldehyde.

The collagen I comparator was a medical-grade, commercially available injectable natural type I collagen gel obtained from Guangzhou Xibei Medical Technology Co., Ltd. (Guangzhou, China) (labeled concentration: 35 mg/mL). It was used directly from the sterile prefilled syringe under aseptic conditions without secondary dilution or chemical modification. A volume of 400 μL per dorsal site was selected because it falls within the approximately 200–500 μL range commonly used for small-animal dermal-filler implantation studies and produces a clearly defined deposit suitable for repeated measurement without excessive local skin tension. This volume also provides a practical local bolus in the approximately 180–200 g rat model for comparison of degradation, volume retention, and host-cell infiltration with typical human facial filler bolus volumes of approximately 0.1–1.0 mL.

$$\mathrm{Volume}\;=\;\frac{4}{3}\;\times \;\pi \;\times \;\frac{1}{2}\;\times \;\mathrm{Length}\;\times \;\frac{1}{2}\;\times \;\mathrm{Width}\;\times \;\frac{1}{2}\times Height$$

Fixed tissues were washed twice in PBS for 5 min, dehydrated sequentially in 50%, 75%, 85%, 95%, and 100% ethanol for 2 h at each concentration, cleared in xylene, infiltrated with paraffin, and embedded. Sections were cut at 8 μm, floated on water at 40–42 °C, mounted on positively charged slides, dried, and stored at 4 °C. Before staining, sections were deparaffinized in xylene and rehydrated through graded ethanol to water. For hematoxylin and eosin staining, sections were stained with hematoxylin for 5 min, differentiated in 1% acid alcohol for 2 s, counterstained with eosin for 10 s, dehydrated, cleared, and mounted. For Masson’s trichrome staining, sections were stained with freshly mixed iron hematoxylin for 1 min, differentiated in 1% acid alcohol for 1 min, and stained with prewarmed Ponceau-acid fuchsin at 60 °C for 4 min. Sections were then treated with phosphomolybdic acid for 3 min without an intervening water wash and stained with aniline blue at 65 °C for 5 s before dehydration, clearing, and mounting. For Sirius Red staining, sections were stained for 1 h, counterstained with hematoxylin for 10 min, washed, dehydrated through graded ethanol, cleared, and mounted. Hematoxylin and eosin- and Masson-stained sections were imaged using a Pannoramic MIDI scanner (3DHISTECH, Budapest, Hungary), while Sirius Red-stained sections were examined under polarized light using a BX51 microscope (Olympus, Tokyo, Japan). Collagen-positive areas and collagen I/III ratios were quantified using ImageJ.

Macrophage-associated inflammation was evaluated by CD68 immunofluorescence staining. Deparaffinized and rehydrated sections were fixed with 4% paraformaldehyde at 37 °C for 10 min, permeabilized with 0.1% Triton X-100 for 5 min, and blocked with 10% FBS in PBS for 30–60 min. Sections were incubated with an anti-CD68 primary antibody (Abcam, Cambridge, UK) overnight at 4 °C in the dark and with the corresponding goat anti-rabbit IgG secondary antibody (Abcam) for 1 h. Nuclei were counterstained with Hoechst 33342 for 7 min. Sections were washed with PBS after each step, imaged by fluorescence microscopy, and analyzed quantitatively using ImageJ.

### 4.7. Statistical Analysis

Data are presented as mean ± standard deviation from three independent replicates (*n* = 3). Statistical analyses were performed using GraphPad Prism 10.1.2. Differences among groups were evaluated by one-way analysis of variance. Statistical significance was defined as *p* < 0.05, with * *p* < 0.05, ** *p* < 0.01, *** *p* < 0.001, and **** *p* < 0.0001.

## Data Availability

The data generated or analyzed during this study are included in this article and are available from the corresponding author upon reasonable request.
